# Delving into the Metabolism of Sézary Cells: A Brief Review

**DOI:** 10.3390/genes15050635

**Published:** 2024-05-17

**Authors:** Carel Cherfan, Alain Chebly, Hamid Reza Rezvani, Marie Beylot-Barry, Edith Chevret

**Affiliations:** 1 BRIC, BoRdeaux Institute of onCology, UMR 1312, Inserm, Université de Bordeaux, 33000 Bordeaux, France; carel.cherfan@u-bordeaux.fr (C.C.); hamid-reza.rezvani@u-bordeaux.fr (H.R.R.); marie.beylot-barry@chu-bordeaux.fr (M.B.-B.); 2 Center Jacques Loiselet for Medical Genetics and Genomics (CGGM), Faculty of Medicine, Saint Joseph University, Beirut P.O. Box 17-5208, Lebanon; alain.chebly@usj.edu.lb; 3 Dermatology Department, Centre Hospitalier Universitaire de Bordeaux, 33075 Bordeaux, France

**Keywords:** Sézary syndrome, cutaneous T cell lymphomas, metabolism, cancer, therapy

## Abstract

Primary cutaneous lymphomas (PCLs) are a heterogeneous group of lymphoproliferative disorders caused by the accumulation of neoplastic T or B lymphocytes in the skin. Sézary syndrome (SS) is an aggressive and rare form of cutaneous T cell lymphoma (CTCL) characterized by an erythroderma and the presence of atypical cerebriform T cells named Sézary cells in skin and blood. Most of the available treatments for SS are not curative, which means there is an urgent need for the development of novel efficient therapies. Recently, targeting cancer metabolism has emerged as a promising strategy for cancer therapy. This is due to the accumulating evidence that metabolic reprogramming highly contributes to tumor progression. Genes play a pivotal role in regulating metabolic processes, and alterations in these genes can disrupt the delicate balance of metabolic pathways, potentially contributing to cancer development. In this review, we discuss the importance of targeting energy metabolism in tumors and the currently available data on the metabolism of Sézary cells, paving the way for potential new therapeutic approaches aiming to improve clinical outcomes for patients suffering from SS.

## 1. Introduction: Primary Cutaneous Lymphomas

Primary cutaneous lymphomas (PCLs) represent a heterogeneous group of lymphoproliferative disorders, characterized by the presence of neoplastic T or B lymphocytes in the skin, with no evidence of extracutaneous disease at the time of the diagnosis. In western countries, approximately 75% to 80% of PCLs originate from T cells (cutaneous T cell lymphomas, CTCLs), while cutaneous B cell lymphomas (CBCLs) account only for 20% to 25% [1]. Mycosis fungoides (MF) and Sézary syndrome (SS) represent the most common forms of CTCL, with MF being the most prevalent type and comprising almost 50% of all PCLs, while SS is less common and constitutes around 2% of CTCLs [1,2,3]. In the following sections, we will mainly focus on SS, the aggressive and leukemic subtype of CTCL. While genetic alterations in SS can affect normal cellular functions, their impact on metabolism, including possible disruptions in metabolic pathways that control cellular energy and nutrient utilization within Sézary cells, remains an area of exploration. Understanding the underlying genetic alterations in SS and their impact on cellular metabolism holds promise for the development of new targeted therapies aimed at correcting metabolic abnormalities and halting disease progression.

### 1.1. Sézary Syndrome

SS is a rare and aggressive subtype of CTCL. It was first described in 1938 by Albert Sézary and Yves Bouvrain as erythroderma associated with superficial lymphadenopathy, along with the presence of “monstrous cells in the dermis and the blood” [4].

SS predominantly affects men, often over the age of 60, and progresses rapidly. The prognosis remains poor, with a 5-year survival rate of 30% [3,5]. Clinical signs of this disease include pruriginous erythroderma, alopecia, palmoplantar keratoderma and onychodystrophy [6]. SS is defined by the triad of erythroderma, generalized lymphadenopathy and the presence of a circulating atypical CD4+ T cell population with cerebriform nuclei (Sézary cells) in the skin, lymph nodes and peripheral blood [1]. Additionally, at least one of the following criteria is also required: an absolute number of Sézary cells ≥ 1000/μL or an expansion of the CD4+ T cell population (CD4/CD8 ratio ≥ 10 or CD4+/CD7− ≥ 30% or CD4+/CD26− ≥ 40%) [1,7,8].

Several biomarkers are identified in Sézary cells, including cell surface markers and genetic alterations. Cell surface markers include TCR-Vβ and other molecules such as the natural cytotoxicity receptor (NCR) NKp46/NCR1, CD85j/Ig-like transcript 2 (ILT2) receptor, CD158k/KIR3DL2 receptor and PD-1, in addition to cutaneous lymphocyte antigen (CLA) and C-C chemokine receptor type 4 (CCR4) [6,9,10,11]. There is evidence suggesting that Sézary cells typically display a central memory T cell phenotype (T_CM_), marked by the presence of CCR7, L-selectin, CD27, CCR4 and CLA [12]. However, other research indicated a broader range of phenotypes beyond the TCM profile, as well as a phenotypic plasticity of these neoplastic cells. This is based on the expression of cell surface markers associated with stem cell memory (T_SCM_), effector memory (T_EM_) and naive or transitional memory T cells (T_TM_) [13].

Genomic alterations reported in SS patients include most frequently TP53 deletion/mutation and other mutations as well as diverse gene expression profiles and miRNA expression [6,14]. Also, it was reported that Sézary cells activate telomerase to maintain their telomeres through a specific methylation pattern on the *hTERT* promoter [15,16]. In addition, studies uncovered a high degree of molecular heterogeneity between patients with SS but also within the malignant T cell population in the same patient [17,18,19]. On one hand, the study of the molecular pathogenesis of the disease contributes to the understanding of Sézary cells’ behavior and response to available treatments but, on the other hand, it highlights the important level of molecular inter- and intra-heterogeneity, thus revealing the complexity in understanding SS and its treatment strategies.

### 1.2. Therapy for Sézary Syndrome

Despite the progress made in implementing therapies for SS, such as targeted therapies, including monoclonal antibodies with long-term responses (i.e., mogamulizumab, directed against CCR4, which has demonstrated a substantial extension in progression-free survival) [20,21,22], none of the existing treatments are curative. The exception is hematopoietic stem cell transplantation (HSCT), which has been associated with significantly longer progression-free survival in patients with advanced-stage CTCLs [23]. However, this curative approach can only be proposed for young and relatively healthy patients with advanced disease with a compatible donor and is associated with a non-negligible morbidity and mortality [6]. Generally, patients are treated using a stage-adapted approach that prioritizes sustaining their quality of life [24]. Moreover, it is recommended that each case of PCL should be brought up at a local or regional multidisciplinary meeting (including members specialized in the field of PCLs) to confirm the diagnosis and therapeutic management of the patient. Handling CTCLs involves three main steps: (1) establishing a definitive diagnosis and classifying the lymphoma according to the EORTC/WHO criteria, (2) determining the stage of the disease and (3) agreeing on a therapeutic approach and reassessing efficacy and side effects in a regular way [25]. Treatment of Sézary syndrome is better managed with systemic therapies (Table 1).

While existing treatments for this lymphoma focus on obtaining a regression of the lesions or slow down its progression, most patients still die from their disease. Consequently, there is an urgent necessity to find alternative effective treatments. Nowadays, metabolic reprogramming is one of the pivotal hallmarks of cancer [26]. Several studies showed that targeting metabolism in cancer has emerged as a promising strategy for novel therapeutic approaches [27]. Hence, exploring the metabolic pathway of Sézary cells could lead to the development of novel therapies with improved outcomes.

**Table 1:** Systemic therapies for SS treatment.

	Comments
Systemic Therapies	
Retinoids (e.g., bexarotene)	Retinoids derive from vitamin A whose function is to interact with nuclear receptors (retinoic acid (RAR) and retinoic X receptor (RXR)). Bexarotene activates RXRs and induces apoptosis and prevents malignant T cell homing to the skin by downregulating CCR4 and E-selectin [3,28].
Interferon α	IFN-α activates CD8+ T cells and NK cells and suppresses cytokine production from malignant lymphoma cells [29].
Extracorporeal photopheresis (ECP)	Patients’ white blood cells are exposed ex vivo to a photosensitizing agent (8-methoxypsolaren) and then to UVA light. Cells are then reinfused in the patient. The purpose is to induce an immune response against malignant T cells [28].
Monoclonal antibodies	Mogamulizumab selectively binds to CCR4—which is highly expressed in malignant T cells—and induces antibody-dependent cellular toxicity, thus destroying tumor cells [20,30]. Lacutamab (IPH4102) binds to CD158k, a cell surface marker aberrantly expressed in patients with SS [31]. IPH4102 is designed to deplete CD158k-expressing cells via antibody-dependent cell cytotoxicity and phagocytosis [22,32].
Antibody–drug conjugate	Brentuximab vedotin (BV) is an anti-CD30 antibody attached to monomethyl auristatin E (MMAE), an antitubulin agent. The binding of BV to CD30 and its internalization will allow MMAE to exert its action and inhibit the assemblage of the microtubules, induce cell cycle arrest and cause cell death due to apoptosis of tumor cells [21,33].
Histone deacetylase inhibitors (HDACis) (e.g., romidepsin)	Histone deacetylases (HDACs) are epigenetic regulators of gene expression. Their inhibitors (HDACis) are reported to induce upregulation of proapoptotic genes, DNA damage and alterations in the assembly of kinetochores [34,35].
Chemotherapy	Single or combined agents can be administered and act via various mechanisms including topoisomerase inhibition, blocking DNA synthesis and interference with essential cellular processes [36].

## 2. Energy Metabolism in Cancer Cells

### 2.1. The Warburg Effect

In the 1920s, Otto Warburg et al. introduced the idea of metabolic reprogramming in cancer research [37]. They showed that cancer cells increased their glucose uptake and metabolized most of it into lactate, even in the presence of oxygen. This phenomenon, also referred to as the “Warburg effect”, leads to ATP synthesis via aerobic glycolysis. Knowing that the energetic yield of glycolysis is very low compared to oxidative phosphorylation (OXPHOS), Warburg hypothesized that cancer cells exhibit an irreversible defect in mitochondrial activity, leading to a metabolic switch towards aerobic glycolysis in order to sustain the cell’s energy demands [37,38].

Since Warburg’s seminal studies, a large number of complex metabolic profiles have been discovered in tumor cells, ranging from highly glycolytic phenotypes [39,40] to completely opposite profiles characterized by increased reliance on OXPHOS [41,42]. Growing evidence indicates that numerous biochemical metabolic pathways undergo reorganization during malignant transformation to fulfill the energy demands of cancer cells and support their biosynthetic fluxes, which are necessary for providing cellular building blocks such as nucleic acids, proteins and membranes, as well as for redox resetting to establish a new redox balance within tumors [39,43,44]. Furthermore, alterations in energy transduction modalities have direct implications for controlling cancer gene expression via transcriptional and epigenetic mechanisms driven by oncometabolites [45]. The pivotal role of mitochondria in tumorigenesis through gene regulation has been evidenced in numerous studies investigating various oncometabolites generated by the tricarboxylic acid (TCA) cycle [39,44] and glutaminolysis [46].

### 2.2. Role of Glycolysis in Cancer

**Figure 1:**
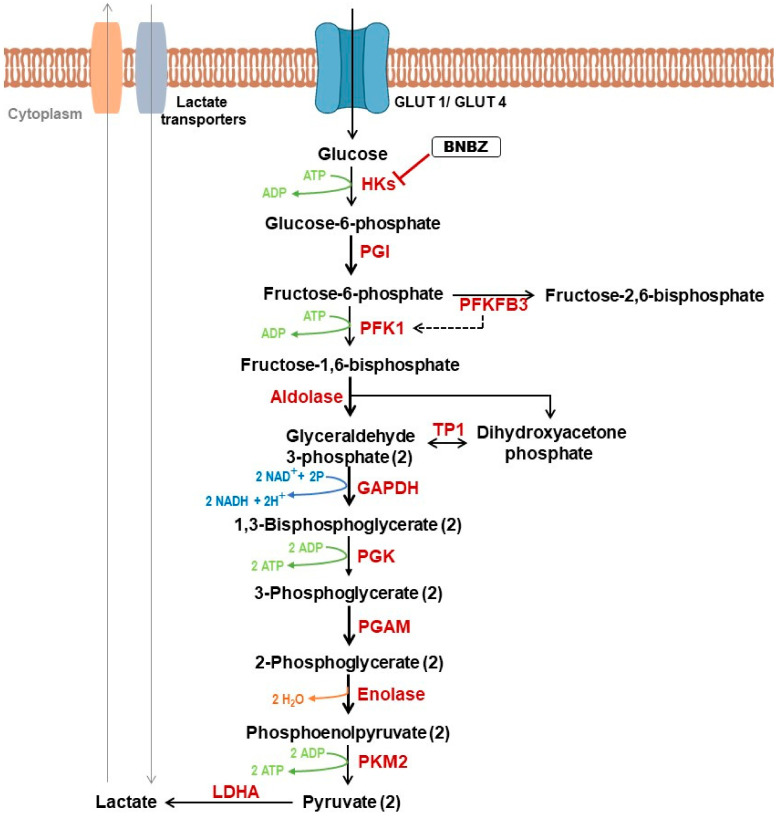
Glycolysis and its inhibitors. Glucose transporter (GLUT); Hexokinase (HK); Phosphoglucose isomerase (PGI); 6-Phosphofructo-2-Kinase/Fructose-2,6-Biphosphatase3 (PFKFB3); Phosphofructokinase 1 (PFK1); Tripsephosphate isomerase (TP1); Glyceraldehyde phosphate dehydrogenase (GAPDH); Phosphoglycerate Kinase (PGK); Phosphoglycerate mutase (PGAM); Pyruvate Kinase M2 (PKM2); Lactate dehydrogenase A (LDHA); Benitrobenrazide (BNBZ).

When oxygen is available, normal cells produce ATP via OXPHOS in the mitochondria. However, cancer cells can prioritize energy production through aerobic glycolysis (Warburg effect). Albeit less efficient than OXPHOS, this metabolic pathway is characterized by high-speed energy production that supports enhanced cell proliferation [47]. In addition to fast ATP production, glycolysis generates many intermediates that can be used as substrates in other anabolic pathways to synthetize cellular components such as lipids, amino acids and nucleotides [40]. Thus, glycolysis fuels anabolic pathways to produce macromolecules that are required for cancer cells’ growth and proliferation. Moreover, glycolysis also contributes to migration of tumor cells. A study conducted by Shiraishi et al. demonstrated that higher glycolysis was associated with increased cytoskeletal remodeling, inducing cellular migration in prostate and breast cancer cells [48]. Furthermore, in the same article, the authors reported that blocking glycolysis impaired cancer cells’ migration, and mitochondrially derived ATP was insufficient to compensate glycolysis [48]. Glycolytic tumor cells convert glucose to lactate (Figure 1) which, once synthesized, is excreted outside of the cell, acidifying the pH in the tumor microenvironment (TME) [49]. Released lactate is reported to contribute significantly to tumor progression via different mechanisms such as angiogenesis, immune escape, cell migration and metastasis [50,51,52].

### 2.3. Role of Mitochondrial Metabolism in Cancer

Numerous findings demonstrated that mitochondrial metabolism is mainly predominant in some types of cancer cells and that this metabolic pathway contributes to cancer progression. Fogal et al. showed that OXPHOS supports tumor maintenance and malignancy. Knockdown of p32 protein, that is frequently overexpressed in some tumors, induced a significant metabolic switch from OXPHOS towards glycolysis in human cancer cells, resulting in impaired cell proliferation *in vitro* and tumor growth *in vivo*. Moreover, high levels of glycolysis in the absence of OXPHOS did not support tumor growth, suggesting that reliance on glycolysis alone may not be very beneficial in cancer cells [53]. Although aerobic glycolysis is known to promote metastasis, studies showed that mitochondrial metabolism also appears to contribute to the migration and invasion process of cancer cells. For instance, silencing of peroxisome proliferator-activated receptor-γ coactivator (*PGC1*-*α*), an inducer of mitochondrial biogenesis, impaired mitochondrial generation and OXPHOS, consequently leading to a decrease in metastatic activity in breast cancer cells [54]. Indeed, mitochondria play a significant role in tumor progression, yet they are also involved in resistance to treatment in cancer cells. For instance, docetaxel-resistant prostate cancer cells were found to have a more efficient respiratory phenotype compared to sensitive cells [55].

### 2.4. Targeting Cancer Metabolism

#### 2.4.1. Targeting Glycolysis

It has been shown that aerobic glycolysis and mitochondrial metabolism can significantly contribute to the malignant phenotype of tumors. Thus, targeting energy metabolism has emerged as a promising strategy for cancer therapy and numerous metabolism-based drugs have been developed or are being tested in clinical trials [27]. Glycolytic phenotype is associated with an upregulation of glycolysis-related enzymes and transporters, making them attractive targets for cancer therapy. Glucose transporters (GLUTs) facilitate glucose uptake and their expression is often increased in different types of tumors. It was reported that treating breast cancer cells and non-small cell lung cancer (NSCLC) cells with antibodies targeting Glut-1 reduced proliferation in human cancer cell lines *in vitro* and enhanced apoptosis induced by chemotherapeutic agents [56]. Glut-1 knockdown has also been shown to suppress glycolysis and proliferation, leading to cell cycle arrest in prostate cancer cell line [57]. Additionally, recent investigations highlighted the significance of palmitoylation for maintaining Glut-1’s localization in the plasma membrane. DHHC9 is the enzyme responsible for this post-translational modification and its knockdown hampers glycolysis, cell proliferation and glioblastoma tumorigenesis *in vivo* [58]. Various studies also focused on targeting enzymes involved in the glycolytic pathway. Hexokinase 2 (HK2) is the first rate-limiting enzyme in glycolysis that catalyzes the conversion of glucose to glucose-6-phosphate (G6P) (Figure 1). This enzyme has been reported to be highly expressed in malignant tumors [59,60]. HK2’s expression was linked to resistance to gemcitabine (GEM) therapy in pancreatic tumor. Knockdown of this enzyme decreased cancer cell proliferation, migration and viability and promoted cell apoptosis *in vitro*. Moreover, reducing HK2 levels resulted in enhanced sensitivity to GEM treatment both *in vitro* and *in vivo* [61]. Indeed, oral administration of benitrobenrazide (BNBZ), an inhibitor that directly binds to HK2 (Figure 1), inhibited tumor growth in mouse xenograft models. *In vitro*, BNBZ is reported to impair glycolysis in cancer cells overexpressing HK2, as well as their proliferation, and to induce their apoptosis [62].

Another rate-limiting enzyme in the glycolytic pathway is phosphosfructokinase-1 (PFK-1). This enzyme catalyzes the phosphorylation of fructose-6-phosphate (F-6-P) to fructose 1,6-bisphosphate (F-1,6bisP). PFK-1 activity is regulated by PFKFB3 which converts F-6-P to fructose-2,6-bisphosphate (F-2,6-BP). PFK-1 can be activated by F-2,6-BP which is itself under the control of PFKFB3 [63] (Figure 1). Increased PFKFB3 activity has been reported in many cancer types, leading to the development of several inhibitors targeting this enzyme with promising results such as impaired migration and invasion capacity of cancer cells [64,65]. Moreover, pyruvate kinase M2 (PKM2) is the final rate-limiting enzyme in glycolysis. It converts phosphoenolpyruvate (PEP) to pyruvate (Figure 1). PKM2 is highly expressed in many cancers and is involved in promoting tumorigenesis, making it an attractive candidate for cancer therapy [66]. Reports showed that the inhibition of PKM2 can suppress proliferation, induce apoptosis and restore drug sensitivity in cancer cells [67,68]. 

#### 2.4.2. Targeting Mitochondrial Metabolism

Several agents have also been developed to target mitochondrial metabolism in cancer cells. They belong to the family of mitocans whose mechanism of action relies on disrupting key actors of mitochondrial metabolism such as the enzymes of the Krebs cycle, the electron transport chain (ETC), proteins belonging to the Bcl-2 family and mitochondrial DNA. Some inhibitors also target anabolic pathways such as glutamine metabolism and fatty acid β-oxidation [38].

**Figure 2:**
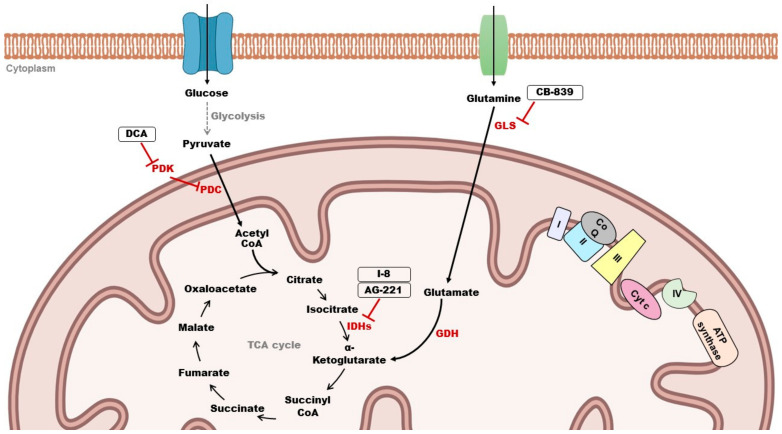
Mitochondrial targets for cancer therapy. Pyruvate dehydrogenase complex (PDC); Pyruvate dehydrogenase kinases (PDKs); Dichloroacetate (DCA); isocitrate dehydrogenase (IDH); Glutaminase (GLS); Glutamate dehydrogenase (GDH); Complexes I, II, III, IV; Coenzyme Q (CoQ); Cytochrome c (Cyt c).

It is known that pyruvate can be converted into acetyl-CoA via decarboxylation that occurs in the mitochondria. This step is a critical linkage between glycolysis and the Krebs cycle. The enzyme complex responsible for this conversion is the pyruvate dehydrogenase complex (PDC) (Figure 2). Pyruvate dehydrogenase kinases (PDKs) act as inhibitors of the PDC and are present in four isoforms (PDK1–4). Their aberrant expression was reported in many types of cancer, making this kinase an attractive target in cancer therapy [63]. Evidence suggests that the inhibition of PDKs can lead to an increase in OXPHOS, therefore limiting the Warburg effect which promotes tumorigenesis [69]. Dichloroacetate (DCA) is an inhibitor of PDKs, and its role has been reported in glioblastoma. Indeed, DCA treatment in glioblastoma cells targeted PDK2 and induced depolarization of mitochondria, apoptosis, inhibition of hypoxia-inducible factor 1-alpha (HIF1-α), activation of p53 and suppression of angiogenesis both *in vitro* and *in vivo* [70]. 

The Krebs cycle, also known as the TCA cycle, represents a series of biochemical reactions taking place in the mitochondrial matrix. It plays an important role in the cell by providing redox balance, energy and intermediates for macromolecule synthesis [71]. Evidence showed that mutations in genes encoding enzymes of the TCA cycle have been associated with cancer progression [72]. For instance, mutations of isocitrate dehydrogenases 1 and 2 (IDH1 and IDH2) are commonly found in hematologic malignancies and solid tumors. These enzymes catalyze the conversion of isocitrate to α-ketoglutarate (αKG) (Figure 2). Mutations in IDH lead to the loss of its typical catalytic activity while acquiring a novel function, the conversion of αKG to the oncometabolite 2-hydroxyglutarate (2-HG) which contributes to tumorigenesis. AG-221 (enasidenib) is an oral inhibitor of mutant IDH2. This inhibitor is currently FDA-approved for the treatment of acute myeloid leukemia (AML) and other solid tumors and has shown promising results [63,73]. I-8 is another inhibitor targeting IDH1 (Figure 2). This compound was found to inhibit the production of 2-HG and suppress histone methylation. In addition, I-8 induced cellular differentiation and decreased tumor stem cell characteristics *in vitro* and *in vivo* [74]. The glutamine pathway has also attracted a lot of attention in cancer therapy. Glutamine serves as an energy source in many cancer types, providing nutrients and precursor molecules for their growth [75]. Glutamine enters the cell via transporters and can be converted into glutamate by glutaminases (GLSs). Then, glutamate is converted to αKG, the intermediate of the TCA cycle, via glutamate dehydrogenase (GDH) (Figure 2). Many studies are currently focusing on inhibiting GLS, which has been proven to have a critical role in various types of cancer [63]. CB-839 is an orally bioavailable inhibitor of GLS in phase I/II clinical trials and has demonstrated antitumor effects in triple-negative breast cancer (TNBC) [76]. Moreover, the ETC in the inner mitochondrial membrane has also been exploited for cancer therapy. It consists of a series of fixed protein complexes (I–IV) and mobile electron carriers (coenzyme Q and cytochrome c) where electrons are transferred, generating a proton gradient used to produce ATP (Figure 2). Inhibitors that are being developed aim to target these different complexes of the inner mitochondrial membrane [77,78].

A growing body of evidence has suggested that metabolism plays a crucial role in the development of resistance and metastasis [79]. Therefore, combining targeted therapies with drugs that exploit vulnerabilities in energy metabolism could lead to significant outcomes in overcoming drug resistance, a prevalent challenge in cancer treatment. For example, melanoma cells that acquired resistance to anti-BRAF treatments largely rely on complex-I-dependent OXPHOS [80,81]. Interestingly, OXPHOS inhibitors or mitochondrial uncouplers have been shown to be sensitive to vemurafenib in BRAF-mutant melanoma cell lines [81]. Similarly, BRAFV600E inhibition was significantly boosted both *in vitro* and in murine models when mitochondrial ETC I was inhibited by antidiabetic biguanides such as metformin [82,83]. However, metformin showed no efficacy in clinical trials, and the supra-pharmacological doses used in pre-clinical stages do not allow for definitive conclusions regarding its potential anticancer mechanism of action [84]. Another mechanism by which BRAF-mutant cells develop resistance to BRAF inhibitors is through upregulation of glutamine uptake and activation of GLS. Consequently, treatment with a GLS inhibitor, such as BPTES, can re-sensitize resistant cells to BRAF inhibition [85]. However, GLS inhibitors have not progressed to the clinical stage due to lack of efficacy, highlighting the need for innovative approaches to advance the field of metabolic inhibition in cancer therapy.

## 3. Metabolism of Sézary Cells: State of the Art

In healthy quiescent CD4+ T cells, metabolism primarily relies on OXPHOS to generate ATP. Upon antigen recognition, metabolism shifts towards an anabolic profile, increasing glucose and amino acid uptake, which better supports clonal expansion and effector phenotype [86]. However, our understanding of the energy metabolism in tumoral T cells, particularly in conditions like SS, remains incomplete.

In a recent study, single-cell RNA sequencing was used to assess the transcriptional profiles of the malignant T cell population in SS samples [87]. This analysis was conducted on four SS blood samples, including two SS patients where both blood and skin samples were analyzed. In the peripheral blood of advanced-stage SS, a heterogenous expansion of T cell clonotypes was identified and each sample exhibited the expansion of a dominant clone as well as of other less abundant ones. Gene expression signatures for each clonotype were established. The results confirm the genetic heterogeneity of Sézary cells, both between patients and within the same patient. Strikingly, the genetic signatures reveal, for the first time, that Sézary cells consistently enhance the expression of OXPHOS genes among a significant number of patients. Additionally, a comparison of T cell clonotype expansion between the blood and the skin of SS patients with advanced disease revealed that one dominant clonotype was expanded in both compartments in each patient. Across all studied patients, this major clonotype revealed common upregulated pathways including OXPHOS.

This finding shows that despite high heterogeneity in malignant SS clones, they were found to upregulate OXPHOS, suggesting a potential homogeneity in this metabolic pathway across SS cases. However, a study based on a larger sample size of patients could be considered to validate this observation. If the data are confirmed, this could be intriguing for therapeutic targeting as they indicate potential vulnerability of Sézary cells that could be exploited for treatment strategies which will focus on disrupting OXPHOS.

Additionally, this major clonotype showed other common upregulated pathways such as HER-2 and sirtuin. Sirtuins control the expression of genes involved in the metabolism via histone deacetylation. They also exert post-translational regulation on various substrates by removing chemical modifications, thereby altering their activity [88]. The sirtuin pathway is poorly characterized in SS, but increasing evidence has confirmed the involvement of sirtuins in cancers with their particular capacity to modulate cellular metabolism [89]. In human breast cancer cell lines, sirtuin 3 (SIRT3), a member of the sirtuin family, acts by indirectly destabilizing HIF1-α, thus inhibiting glycolysis. This protein appears to be downregulated in human breast cancer, and this is associated with an increased expression of HIF1-α target genes [90]. Moreover, knockdown of SIRT3 in a metastatic colon cancer cell line leads to decreased mitochondrial biogenesis and mitochondrial dysfunction, consequently affecting cellular viability [91]. In light of this, elucidating the potential involvement of sirtuins in SS could aid in the development of targeted therapeutic interventions.

ATP metabolism was described to have an influence on the immune response of T cells derived from patients with SS [92]. It is known that CD39 and CD73 are ectonucleotidases catalyzing the conversion of ATP or ADP to AMP, and AMP to adenosine (ADO), respectively [93]. Increased concentrations of ADO metabolite in the TME are reported to induce immunosuppressive mechanisms [94]. Indeed, it has been reported that aberrant expression of CD39 and CD73 in circulating or skin-homing CD4+ T cells of SS patients can affect the metabolism of ATP as well as the production of ADO, thus impacting immune responses [92]. In this study, patients with higher levels of CD39+ cells (CD39^high^) demonstrated an increased activity in regard to hydrolyzing ATP into AMP. Conversely, patients with higher levels of CD73+ cells (CD73^high^) exhibited strong AMP metabolism, generating high concentrations of ADO. Moreover, coculture experiments revealed that CD39^high^ cells produced large amounts of ADO from ATP. This overproduction of ADO was not detected in CD73^high^ cells. Altogether, these results suggest that CD39^high^ cells, alongside surrounding cell types that express CD73, can create an environment rich in ADO to induce immunosuppression [92].

A recent study published by Wartewig et al. showed that the inactivation of *Pdcd1*, which encodes the inhibitory receptor programmed death-1 (PD-1) known for inhibiting the antitumor immune response, enforces T cells with oncogenic signaling to adjust their metabolism towards aerobic glycolysis [95,96]. This was confirmed in both genetic mouse models and blood samples of patients with CTCL, diagnosed with either SS or leukemic MF. RNA-seq analysis showed enhanced activity of the PI3K-AKT-mTOR pathway, enforced HIF1-α activity and increased activity of glycolysis genes in *Pdcd1*-mutant lymphomas, compared to wild type *Pdcd1*. These results uncover a vulnerability in CTCL-deficient in *Pdcd1*, that can further be exploited in the implementation of novel therapies that involve targeting energy metabolism.

Another study also described the involvement of the PI3K/AKT/mTOR signaling pathway in Sézary cells [97]. They identified that Sézary cells residing in the skin display a higher proliferation rate compared to those circulating in the blood. Interestingly, skin-resident Sézary cells exhibit a higher activation of mTORC1/mTORC2 compared to blood-circulating cells. This activation is influenced by SDF-1 and CCL21 chemokines, triggering mTORC1 signaling, which is known to be a central regulator of cell metabolism. Additionally, genetic alterations within the PI3K/AKT/mTOR cascade in Sézary cells strongly impact their cellular energy. For instance, the authors reported that loss of PTEN and LKB1, which attenuate the activation of mTORC1 under normal conditions, can lead to constitutive activation of mTORC1 in Sézary cells [98,99]. This induces a metabolic shift towards aerobic glycolysis. These results clearly show that metabolism might play a role in SS. Hence, conducting a more exhaustive study would be necessary to validate these observations.

All together, these findings emphasize the importance of considering metabolic reprogramming in the setting of SS.

## 4. Conclusions and Perspectives

In conclusion, available data regarding Sézary cells’ metabolism remain very scarce. A better understanding of the energy metabolism of these cells is primordial and could lead to a better understanding of the pathogenesis of SS and ideally a better therapeutic management of SS patients in the era of precision medicine and targeted therapies. Hence, further research is needed to decipher metabolic pathways behind Sézary cells’ survival. This knowledge will allow us to disrupt Sézary cells’ energy balance and to propose effective and well-tolerated therapeutic strategies, in combination with current treatments or in addition to the treatments offered today.

## Data Availability

No new data were created or analyzed in this study. Data sharing is not applicable to this article.
